# The causal association between sleep traits and osteoarthritis traits: evidence from bidirectional mendelian randomization

**DOI:** 10.1093/hmg/ddag043

**Published:** 2026-06-01

**Authors:** Shibo Chen, Lorraine Southam, Ana Luiza Arruda, Eleftheria Zeggini

**Affiliations:** Institute of Translational Genomics, Computational Health Center, Helmholtz Zentrum München – German Research Center for Environmental Health, Ingolstädter Landstraße 1, Bavaria, Neuherberg 85764, Germany; Munich Medical Research School (MMRS), Faculty of Medicine, Ludwig-Maximilians-Universität München, Geschwister-Scholl-Platz 1, Bavaria, Munich 80539, Germany; Institute of Translational Genomics, Computational Health Center, Helmholtz Zentrum München – German Research Center for Environmental Health, Ingolstädter Landstraße 1, Bavaria, Neuherberg 85764, Germany; Institute of Translational Genomics, Computational Health Center, Helmholtz Zentrum München – German Research Center for Environmental Health, Ingolstädter Landstraße 1, Bavaria, Neuherberg 85764, Germany; Institute of Metabolic Science (IMS) Epidemiology, University of Cambridge School of Clinical Medicine, Box 285 Institute of Metabolic Science, Cambridge Biomedical Campus, Cambridge, CB2 0QQ, United Kingdom; Institute of Translational Genomics, Computational Health Center, Helmholtz Zentrum München – German Research Center for Environmental Health, Ingolstädter Landstraße 1, Bavaria, Neuherberg 85764, Germany; Technical University of Munich (TUM), TUM University Hospital, TUM School of Medicine and Health, Ismaninger Str. 22, Bavaria, 81675 Munich, Germany

**Keywords:** osteoarthritis, mendelian randomization, sleep disorder, circadian rhythm, genome-wide association study

## Abstract

Sleep disorder is associated with risk of osteoarthritis, yet their causal association has not been fully understood. This study aims to evaluate the causal association between sleep traits and osteoarthritis, by performing two sample Mendelian randomization (MR) leveraging the currently largest genome-wide association study (GWAS) summary statistics for sleep traits and osteoarthritis traits. We demonstrate that insomnia has a risk-increasing causal effect on osteoarthritis at any site, hip osteoarthritis, and hip and/or knee osteoarthritis, corresponding to approximately 60%, 100%, and 50% higher odds of disease, respectively, with the strongest effect observed for hip osteoarthritis. Conversely, genetic predisposition to osteoarthritis at any site was associated with a modest 4% increase in the odds of insomnia. These findings reveal a bidirectional causal relationship between insomnia and osteoarthritis-related traits, underscoring the critical interplay between sleep health and joint disease, and suggesting opportunities for preventive and therapeutic strategies that align with the body’s intrinsic timing systems.

## Introduction

Osteoarthritis is the most common degenerative joint disease and a leading cause of pain and disability worldwide. It is characterized by the progressive breakdown of articular cartilage, synovial inflammation, and structural changes in subchondral bone [1]. The pathogenesis of osteoarthritis is multifactorial, involving aging, genetic predisposition, obesity, mechanical overload, and metabolic alterations, all of which interact to disrupt cartilage homeostasis and accelerate joint degeneration [2–4]. Despite advances in understanding disease mechanisms, there is currently no curative therapy for osteoarthritis, and existing treatments are largely symptomatic [5], highlighting the urgent need to identify novel biological pathways and modifiable risk factors that can be targeted for prevention and disease modification.

One emerging area of interest is the role of sleep disorder in joint biology and osteoarthritis development. Sleep disorders can be attributed to disturbances in circadian rhythm, which consists of endogenous, near-24-hour cycles that regulate a wide range of physiological processes, including metabolism, immune function, and tissue repair, through the activity of central and peripheral molecular clocks [6–8]. At the cellular level, circadian rhythms are maintained by transcriptional-translational feedback loops of core clock genes, such as *BMAL1, CLOCK, PER*, and *CRY* [9]. These clock genes synchronize cellular activity with environmental cues, primarily the light–dark cycle, to optimize biological function [10]. When circadian rhythms are disrupted, whether due to aging, environmental factors, or behavioral influences such as irregular sleep or night shift work, physiological processes can become dysregulated, leading to increased susceptibility to chronic diseases.

Molecular evidence suggests a critical role for circadian clocks in maintaining cartilage integrity. Chondrocytes harbor intrinsic circadian clocks that regulate the rhythmic expression of genes involved in extracellular matrix metabolism, inflammation, and oxidative stress responses [11]. Disruption of core clock genes, such as *BMAL1*, in experimental models leads to loss of rhythmicity, impaired cartilage homeostasis, and osteoarthritis-like degeneration [12]. Similarly, the circadian regulator *NR1D1* (Rev-Erbα) has been shown to influence cartilage metabolism, with its dysregulation exacerbating osteoarthritis pathology [13]. Furthermore, aging and chronic inflammation weaken circadian amplitude in cartilage, while experimental circadian disruption, such as repeated light–dark cycle shifts, facilitates cartilage degeneration in animals [14]. Hormones under circadian control, including melatonin and cortisol, also modulate chondrocyte activity and inflammatory signaling, providing additional mechanistic links between circadian physiology and joint health [15, 16]. Genetic studies have revealed that a number of potential effector genes associated with osteoarthritis are involved in the biological processes of the circadian clock [17].

Epidemiological studies support these molecular insights by demonstrating associations between sleep disorder and osteoarthritis risk. Data from large population-based cohorts, including the UK Biobank and the Dongfeng–Tongji cohort, show that night shift work and rotating shift schedules are linked to a higher risk of knee and lower-extremity osteoarthritis, with longer shift-work duration conferring greater risk [18, 19]. Sleep-related traits also appear to be important: both very short (<6 h) and very long (≥9 h) sleep duration are associated with increased osteoarthritis prevalence, while poor sleep quality and frequent restless nights predict incident knee osteoarthritis in longitudinal studies [20, 21]. An observational study investigating the healthy lifestyle and risk of osteoarthritis, also demonstrated that a 7–8 hours’ sleep duration is the most protective lifestyle for osteoarthritis [22]. Moreover, sleep disorders such as sleep apnea and hypersomnia have been linked to higher overall osteoarthritis risk [23]. A Mendelian randomization study indicated that sleep disturbance, including insomnia and short sleep duration, had a risk-increasing causal effect on osteoarthritis at any site [24].

Taken together, these findings suggest that sleep disorder may play an important role in osteoarthritis. Nevertheless, the causal relationship between sleep traits and osteoarthritis traits remains insufficiently understood. Mendelian randomization (MR) is an analytical approach that uses genetic variants as instrumental variables to infer potential causal relationships between exposures and outcomes. Because genetic variants are randomly allocated at conception according to Mendel’s laws, MR analyses are less susceptible to confounding and reverse causation that often affect conventional observational studies. Valid MR inference relies on three key assumptions: (i) the genetic variants are robustly associated with the exposure, (ii) the variants are not associated with confounders of the exposure-outcome relationship, and (iii) the variants influence the outcome only through the exposure. In this study, we aim to elucidate the interplay between sleep traits and osteoarthritis risk applying MR, with the potential not only to advance our understanding of osteoarthritis pathogenesis but also to inform novel therapeutic strategies, such as chronotherapy and lifestyle interventions aimed at restoring healthy sleep behavior and circadian alignment and improving joint health.

## Results

### Causal effects of sleep traits on osteoarthritis traits

**Figure 1:**
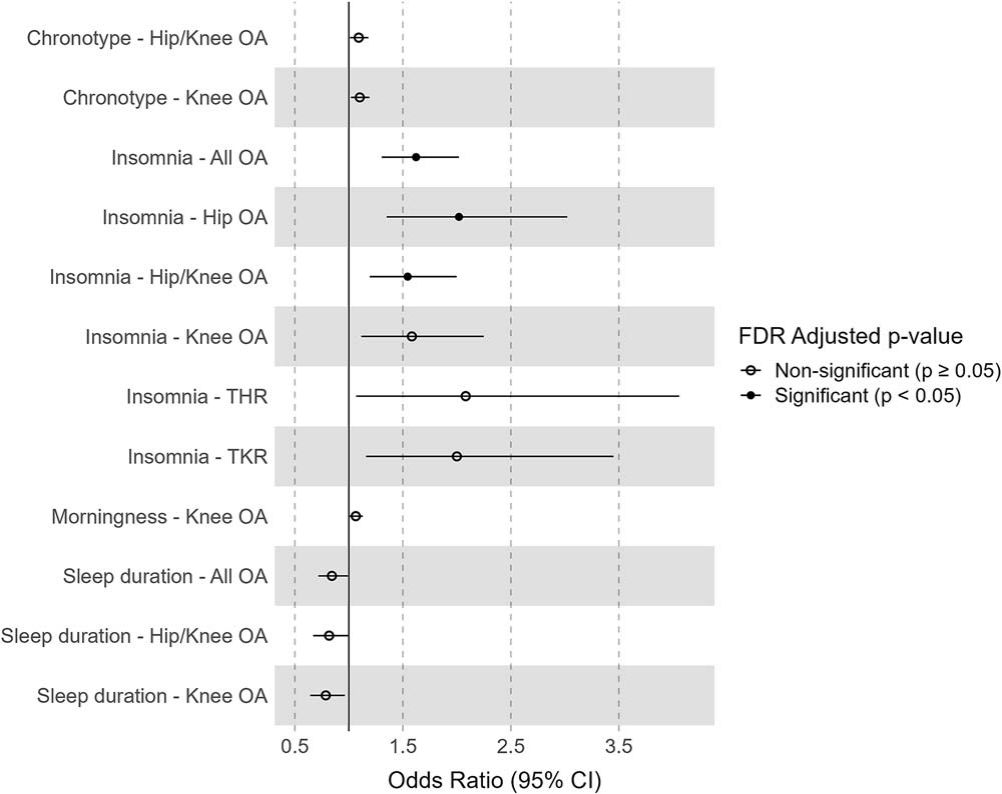
The effect of sleep traits on osteoarthritis traits. The forest plot shows the nominal significant (P-value < 0.05) causal effects between sleep traits and osteoarthritis. Circle mark represents non-significant associations after multiple testing correction (FDR ≥ 0.05), while the filled dot represents significant associations (FDR < 0.05).

For the sleep traits, after filtering and clumping, we included 23, 83, 52, 37 SNPs for insomnia, chronotype, morningness and sleep duration, respectively (Supplementary Table 1). Among sleep traits, genetic predisposition to insomnia demonstrated robust risk-increasing causal effects on osteoarthritis at any site (OR (95% CI) = 1.62 (1.30 to 2.02), P_IVW_ = 1.40 × 10^−5^), hip osteoarthritis (OR (95% CI) = 2.02 (1.35 to 3.02), P_IVW_ = 6.35 × 10^−4^), and hip/knee osteoarthritis (OR (95% CI) = 1.54 (1.19 to 2.00), P_IVW_ = 9.32 × 10^−4^), which remained significant after multiple testing correction (FDR_IVW_ = 3.92 × 10^−4^, 8.69 × 10^−3^, 8.69 × 10^−3^, respectively) (Fig. 1). Insomnia also showed a nominal causal effect on knee osteoarthritis (OR (95% CI) = 1.58 (1.11 to 2.25), P_IVW_ = 0.010), total knee replacement (TKR) (OR (95% CI) = 2.00 (1.16 to 3.45), P_IVW_ = 0.013) and total hip replacement (THR) (OR (95% CI) = 2.08 (1.07 to 4.06), P_IVW_ = 0.031); however, these are not significant after multiple testing correction (Supplementary Table 2). In contrast, increased sleep duration showed a causal effect on reduced risk of osteoarthritis at any site (OR (95% CI) = 0.84 (0.72 to 0.99), P_IVW_ = 0.039), knee osteoarthritis (OR (95% CI) = 0.79 (0.64 to 0.96), P_IVW_ = 0.021), and hip/knee osteoarthritis (OR (95% CI) = 0.82 (0.67 to 1.00), P_IVW_ = 0.047). After multiple testing correction, these effects are not significant (FDR_IVW_ = 0.099, 0.083, 0.11 respectively) (Fig. 1). To compare the consistency between categorical scale chronotype and binary chronotype, we performed analyses on categorical chronotype and morningness. A categorical scale of chronotype showed a risk-increasing causal effect on knee osteoarthritis (OR (95% CI) = 1.10 (1.02 to 1.19), P_IVW_ = 0.016) and hip/knee osteoarthritis (OR (95% CI) = 1.09 (1.01 to 1.18), P_IVW_ = 0.029) (Fig. 1), while a morning chronotype was also causally associated with knee osteoarthritis (OR (95% CI) = 1.064 (1.00 to 1.13), P_IVW_ = 0.039), and although not significant but consistently associated with increased risk of knee osteoarthritis (OR (95% CI) = 1.06 (0.99 to 1.12), P_IVW_ = 0.075) (Fig. 1) (Supplementary Table 2). These associations were also not significant after multiple testing correction (FDR_IVW_ = 0.075, 0.098, 0.099, respectively) (Fig. 1).

### Sensitivity analysis

Sensitivity analyses, including weighted median (WM), MR-Egger, pleiotropy test and heterogeneity tests, were conducted to evaluate the robustness of the causal estimates and to assess potential violations of MR assumptions. The results were interpreted primarily based on concordance in effect direction across methods, rather than statistical significance, due to the limited power of some sensitivity analyses. The WM analysis corroborated the findings from the main analysis described above using the random-effects inverse variance weighted (IVW) analysis, demonstrating consistent association direction between insomnia and risk of osteoarthritis at any site (OR (95% CI) = 1.48 (1.18 to 1.85), P_WM_ = 6.76 × 10^−4^), hip osteoarthritis (OR (95% CI) = 2.57 (1.67 to 3.91), P_WM_ = 1.44 × 10^−5^), and hip/knee osteoarthritis (OR (95% CI) = 1.41 (1.02 to 1.95), P_WM_ = 0.036) (Supplementary Table 1). The MR-Egger analysis also showed the same direction of causal effects for insomnia on the increased risk of osteoarthritis at any site (OR (95% CI) = 1.58 (0.82 to 3.04), P_MR-Egger_ = 0.19), hip osteoarthritis (OR (95% CI) = 4.13 (1.34 to 12.69), P_MR-Egger_ = 0.022), and hip/knee osteoarthritis (OR (95% CI) = 1.83 (0.85 to 3.95), P_MR-Egger_ = 0.14), but the associations are not significant. MR-Egger pleiotropy test showed no significant evidence of horizontal pleiotropy for the associations between insomnia and risk of osteoarthritis at any site (P _pleiotropy test_ = 0.93), hip osteoarthritis (P _pleiotropy test_ = 0.20) and hip/knee osteoarthritis (P _pleiotropy test_ = 0.65) (Supplementary Table 1). The heterogeneity test results indicate that significant heterogeneity was observed for the association between insomnia and risk of osteoarthritis at any site (I^2^ = 52.0%) and hip osteoarthritis (I^2^ = 53.3%), while low heterogeneity was observed for the association between insomnia and risk of hip/knee osteoarthritis (I^2^ = 34.4%) (Supplementary Table 1). Additionally, to assess potential pleiotropy, we evaluated associations of the instrumental SNPs with negative control outcomes (hair color and handedness) and potential confounders including body mass index (BMI), type 2 diabetes (T2D), systolic blood pressure (SBP), diastolic blood pressure (DBP), coronary artery disease (CAD) and joint pain. We found no evidence that instrumental SNPs were associated with hair colour (dark brown or black) or right-handedness at genome-wide significance (Supplementary Table 3, Supplementary Table 4, Supplementary Table 5). However, we identified several SNPs that were genome-wide significantly associated with potential genetic confounders (Supplementary Table 6, Supplementary Table 7, Supplementary Table 8, Supplementary Table 9, Supplementary Table 10, Supplementary Table 11). By excluding these SNPs, we performed a sensitivity MR analysis of which the results shown consistent findings with the main analyses indicating that insomnia showing a robust significant association with the risk of overall osteoarthritis, as well as hip, knee, and hip/knee osteoarthritis (Supplementary Table 12). In addition, the Steiger directionality test suggest that the directions of causal association for insomnia on osteoarthritis at any site, hip osteoarthritis as well as hip and/or knee osteoarthritis are correct (P directionality test = 3.28 × 10–55, P directionality test = 3.09 × 10–54, P directionality test = 3.22 × 10–159, respectively) (Supplementary Table 13). Moreover, Steiger filtering results showed that the causal association between insomnia and osteoarthritis at any site (FDR = 1.05 × 10^−8^) as well as hip osteoarthritis (FDR = 1.54 × 10^−3^) remain significant (Supplementary Table 14).

### Reverse direction MR

**Figure 2:**
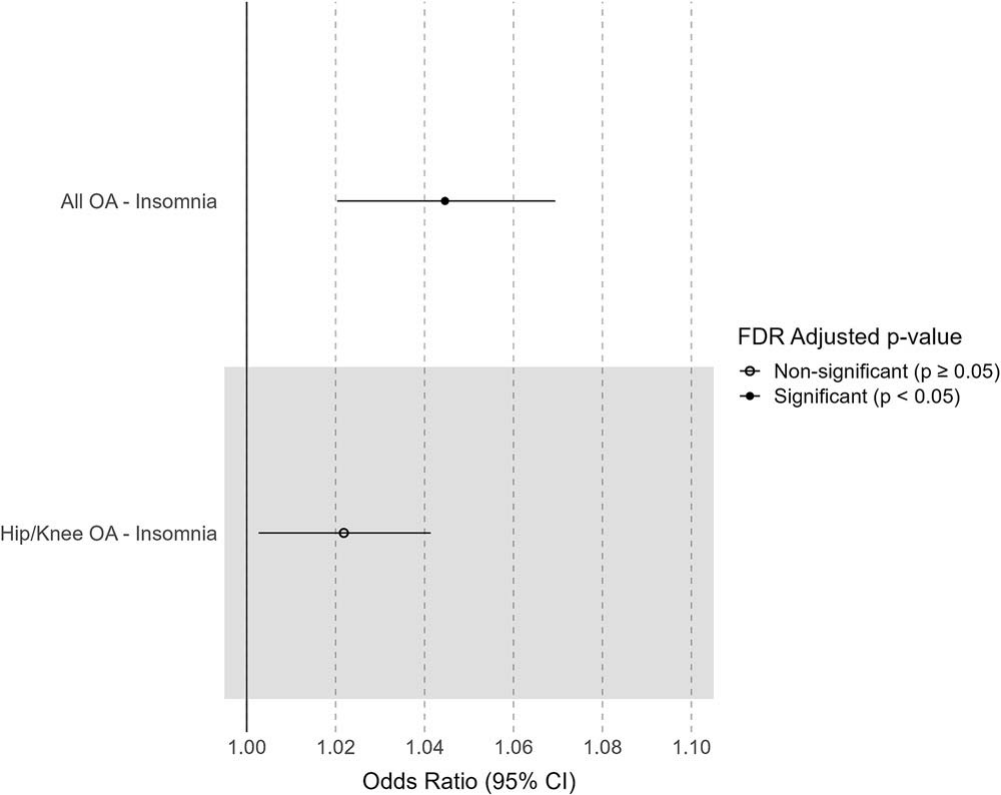
The effect of osteoarthritis traits on sleep traits. The forest plot shows the nominal significant (P-value < 0.05) causal effects between osteoarthritis and sleep traits. Circle mark represents non-significant associations (FDR ≥ 0.05), while the filled dot represents significant associations (FDR < 0.05).

We found evidence of a risk-increasing causal effect of genetic predisposition to osteoarthritis at any site (OR (95% CI) = 1.04 (1.02 to 1.07), P_IVW_ = 2.68 × 10^−4^) as well as to hip/knee osteoarthritis (OR (95% CI) = 1.02 (1.00 to 1.04), P_IVW_ = 0.025) on insomnia. After correcting for multiple testing, only the effect of genetic predisposition to osteoarthritis at any site on insomnia risk remained significant (FDR = 7.51 × 10^−3^) (Fig. 2, Supplementary Table 15). The risk-increasing effect direction of genetic predisposition to osteoarthritis at any site on insomnia risk was corroborated by the WM and MR-Egger methods.

### MR-PRESSO analysis

**Figure 3:**
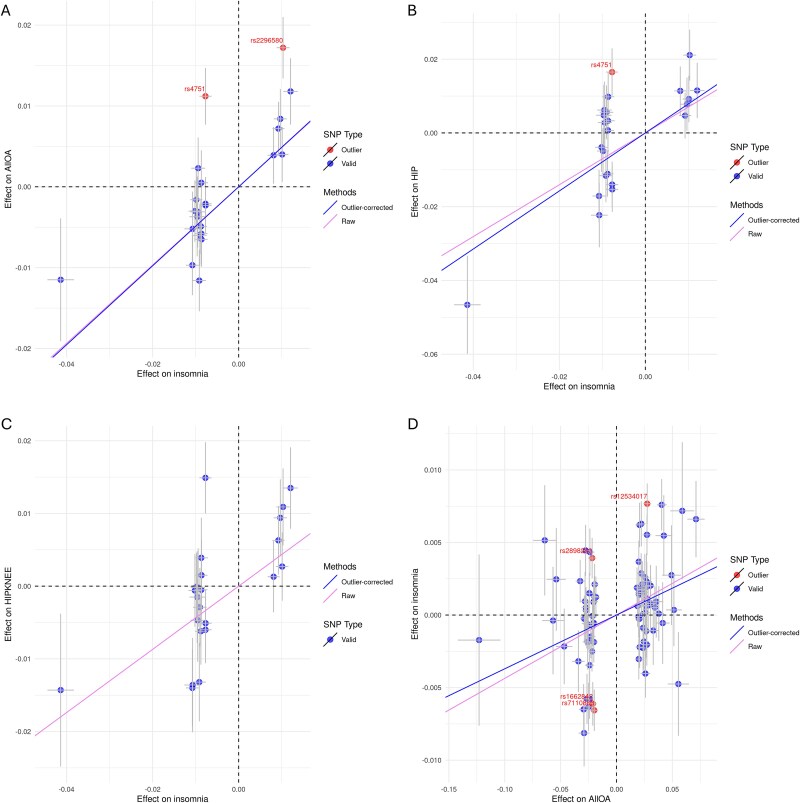
The scatter plots show bi-directional effect between sleep traits and osteoarthritis before and after outlier correction (MR-PRESSO). The blue dots represent valid SNPs, while the red dots represent outlier SNPs. The red line represents fitted estimation by all SNPs including the outliers, while the blue line represents fitted estimation by valid SNPs excluding outliers.

We also used the approach of MR pleiotropy residual sum and outlier method (MR-PRESSO) to validate the IVW MR results. The results of MR-PRESSO demonstrate that there are possible biases from horizontal pleiotropy that could influence the causal effect of genetic predisposition to insomnia on osteoarthritis at any site and hip osteoarthritis (P_MR-PRESSO global test_ = 0.003 and P_MR-PRESSO global test_ = 0.002 for osteoarthritis at any site and hip osteoarthritis, respectively), but not of genetic predisposition to insomnia on hip and/or knee osteoarthritis (P_MR-PRESSO global test_ = 0.062) (Fig. 3A, B, and C). After correcting for the detected horizontal pleiotropy effects using the MR-PRESSO method, the causal effect of genetic predisposition to insomnia on osteoarthritis at any site and hip osteoarthritis show consistent direction of effect (risk-increasing) and remain significant (OR = 1.63 (1.34 to 1.98), P _correction_ = 2.18 × 10^−6^ and OR = 2.20 (1.43 to 3.37), P _correction_ = 3.40 × 10^−4^ for all osteoarthritis and hip osteoarthritis, respectively) (Fig. 3A, and B). The MR-PRESSO distortion test indicated that there is no significant difference for the casual association after adjustment for outliers (P _MR-PRESSO distortion test_ = 0.96 and P _MR-PRESSO distortion test_ = 0.67 for osteoarthritis and at any site hip osteoarthritis, respectively) (Supplementary Table 16). In the reverse direction, MR-PRESSO analyses demonstrated that there is possible bias from horizontal pleiotropy that could influence the identified causal effect of genetic predisposition to osteoarthritis at any site on insomnia risk (P_MR-PRESSO global test_ < 0.001). After correcting for the detected horizontal pleiotropy effects, the causal effect of genetic predisposition to osteoarthritis at any site on insomnia risk remained significant (OR = 1.038 (1.015 to 1.062), P _correction_ = 1.19 × 10^−3^) (Fig. 3D). The MR-PRESSO distortion test showed no significant difference for the casual association between genetic predisposition to osteoarthritis at any site and insomnia risk after adjustment for outliers (P _MR-PRESSO distortion test_ = 0.45) (Supplementary Table 17).

### Mediation analyses by multivariable MR (MVMR)

To investigate whether there are any factors mediate the association between insomnia and risk of osteoarthritis, we performed MVMR by including BMI and major depression disorder (MDD) as exposure with insomnia on osteoarthritis traits. The results suggest that BMI may partially mediate the association between insomnia and hip osteoarthritis, as well as hip/knee osteoarthritis (OR = 1.37 (0.94 to 2.00), P _correction_ = 0.15 and OR = 1.17 (0.92 to 1.50), P _correction_ = 0.27 for hip osteoarthritis and hip/knee osteoarthritis, respectively), while the association between insomnia and overall osteoarthritis remained significant after adjustment (OR = 1.41 (1.13 to 1.75), P _correction_ = 9.65 × 10^−3^). In contrast, MDD did not appear to mediate the effect of insomnia on osteoarthritis outcomes (Supplementary Table 18).

### MR on accelerometer-derived data

To objectively evaluate the role of sleep behaviour in osteoarthritis risk, we performed an additional MR analysis using accelerometer-derived sleep phenotypes. Overall, most of the associations did not reach statistical significance after correction for multiple testing (FDR > 0.05). For the number of sleep episodes, a proxy for sleep fragmentation, the direction of effect was consistently positive across all osteoarthritis outcomes, suggesting that greater sleep fragmentation may be associated with higher osteoarthritis risk, although these associations did not reach nominal significance. The most significant association for the number of sleep episodes was observed on osteoarthritis at any site (OR (95% CI) = 1.13 (0.99 to 1.29), P_IVW_ = 0.069, FDR = 0.337). For sleep midpoint, a nominally significant association was observed with knee osteoarthritis (OR (95% CI) = 0.52 (0.36 to 0.74), P_IVW_ = 0.0004, FDR = 0.018), and hip and/or knee osteoarthritis (OR (95% CI) = 0.65 (0.46 to 0.93), P_IVW_ = 0.017, FDR = 0.248). For diurnal inactivity, nominally significant associations were found with total hip replacement (OR (95% CI) = 1.67 (1.12 to 2.48), P_IVW_ = 0.011, FDR = 0.248) and total joint replacement (OR (95% CI) = 1.52 (1.06 to 2.18), P_IVW_ = 0.021, FDR = 0.248), though neither survived FDR correction. No consistent associations were observed for L5 timing, M10 timing, sleep duration, or sleep efficiency across osteoarthritis outcomes (Supplementary Table 19).

## Discussion

In this bidirectional MR analysis, we examined the potential causal relationship between sleep traits and osteoarthritis traits. Specifically, we investigated whether genetic predisposition to altered sleep traits influence the risk of osteoarthritis, and, conversely, whether genetic liability to osteoarthritis influences insomnia. Our findings provide novel insights into the interplay between sleep traits and joint health and highlight the complexity of this relationship.

The causal effects of insomnia on osteoarthritis risk suggest that genetic predisposition to insomnia has a risk-increasing causal effect on osteoarthritis at any site, hip osteoarthritis, and hip/knee osteoarthritis. Several studies have reported that sleep disorder can be attributed to the disruption of circadian rhythm which has been implicated in a range of physiological processes relevant to osteoarthritis pathogenesis, including cartilage metabolism, inflammatory responses, and pain perception [6, 7, 25]. Animal and *in vitro* studies have shown that core clock genes, such as *BMAL1* and *CLOCK*, are expressed in chondrocytes and synovial cells, influencing matrix turnover and inflammatory signaling [9]. Our MR results support the hypothesis that insomnia may contribute to osteoarthritis development, possibly through mechanisms involving altered chondrocyte homeostasis, increased oxidative stress, and systemic metabolic changes. Additionally, the results of MVMR indicate that BMI may be the mediator driving the development of osteoarthritis at weight bearing joint (hip and hip/knee). We did not find evidence of a causal effect between osteoarthritis and sleep duration or chronotype, possibly due to the nature of these phenotypes. Insomnia reflects difficulties in initiating and maintaining sleep, directly indicating circadian rhythm disturbance. In contrast, chronotype captures an individual’s sleep timing, and sleep duration reflects the length of the sleep period; neither of these measures necessarily indicate whether sleep is restful or disrupted. Additionally, in animal models investigating the link between circadian rhythm and osteoarthritis, circadian disruption is typically induced by altering the light–dark cycle, for example, by imposing constant darkness or reversing the cycle [26, 27]. Such manipulations resemble the disturbance of circadian rhythm observed in insomnia, but not the traits of chronotype or sleep duration.

We found evidence that genetic liability to osteoarthritis causally affects insomnia risk. This may indicate that genetic variants associated with osteoarthritis risk directly affect the central biological clock. Recent studies showed that biochemical and biomechanical cues from the extracellular matrix, for instance, pathways mediated by TGFβ, can regulate circadian clocks [28, 29]. This evidence suggests that sleep, potentially driven by circadian rhythm disruption, may influence each other with osteoarthritis mutually, such that improving one could benefit the other and contribute to a positive cycle of health. However, it is important to consider that the experience of chronic pain, reduced mobility, and psychological comorbidities in osteoarthritis patients can secondarily influence sleep timing, quality, and activity patterns [30, 31]. Such effects are more likely to be mediated by environmental and behavioral pathways rather than by the genetic architecture of osteoarthritis itself. Therefore, while our findings support a causal effect of genetic predisposition to osteoarthritis on insomnia risk, they should be interpreted with caution, and further studies are needed to identify potential mediating factors underlying this relationship.

The findings in this study are consistent with epidemiological evidence linking shift work and sleep disturbances to a higher risk of musculoskeletal disorders and more severe symptoms [18, 32, 33]. Moreover, our findings of causal effects between insomnia on osteoarthritis traits are consistent with the evidence from another MR study that indicates a causal effect of insomnia genetic predisposition on risk of osteoarthritis at any site, hip/knee osteoarthritis, and hip osteoarthritis [24]. While consistent with Ni et al. (2022), our study offers several important advances. First, we utilized the latest and largest GWAS summary statistics from the Genetics of Osteoarthritis 2.0 (GO 2.0) consortium (53% of the identified independent signals are previously unreported) without overlapping individuals, substantially increasing the number of available genetic instruments and enhancing the statistical power and robustness of our causal estimates. Second, our bidirectional MR framework extends beyond unidirectional analysis by additionally examining whether osteoarthritis causally influences insomnia, revealing a mutual causal relationship between insomnia and osteoarthritis that has not been previously reported and carries meaningful clinical implications. Third, our study extends beyond the investigation of insomnia and sleep duration to include chronotype and morningness as potential causal risk factors for osteoarthritis; these findings provide important boundary conditions for understanding which sleep phenotypes are causally relevant to osteoarthritis risk. Fourth, we extended our analysis by incorporating accelerometer-derived sleep phenotypes as objective measures of sleep behaviour, providing more direct evidence that objectively measured sleep disruption. Finally, while Ni et al. (2022) reported a weak adverse association between short sleep duration and osteoarthritis risk, our study found no significant causal association between sleep duration and osteoarthritis risk after correction for multiple testing, suggesting that this association may not be robust and warrants cautious interpretation.

In clinical practice, the health management of osteoarthritis primarily focuses on maintaining a healthy weight and engaging in regular physical activity [34]. However, healthy sleep patterns are not routinely emphasized as part of osteoarthritis care. Our findings highlight the potential causal role of sleep disorder in osteoarthritis pathogenesis suggesting that interventions targeting sleep health and circadian health, such as optimizing sleep quality, light exposure, and activity scheduling, could be particularly beneficial for individuals at elevated risk of developing osteoarthritis. Furthermore, reducing osteoarthritis risk may in turn alleviate insomnia symptoms, creating a positive feedback loop that lowers the risk of both conditions, as supported by our evidence of a reverse directional effect of osteoarthritis on insomnia. Moreover, therapeutic strategies aimed at modulating circadian rhythm, such as the use of melatonin [35, 36], warrant further investigation as potential assistive approaches for both alleviating symptoms in osteoarthritis patients and preventing disease onset in high-risk populations. Future studies integrating genomic, transcriptomic, and longitudinal phenotyping approaches may help to clarify the molecular pathways linking sleep traits to joint health and to identify subgroups of patients who may benefit most from chronobiology-informed interventions.

Our findings should be interpreted in light of several limitations. First, although MR minimizes confounding and reverse causation, residual pleiotropy cannot be completely excluded. Second, the genetic instruments used for sleep traits and osteoarthritis were derived primarily from populations of European ancestry, which may limit generalizability to other ancestry groups. Third, sleep behavior is a complex, multidimensional construct, and the genetic proxies used here may capture only specific aspects rather than the full spectrum of sleep physiology. Fourth, sleep duration was modeled as a continuous variable assuming a linear relationship with the outcome. However, observational evidence suggests potential U-shaped associations [21, 37], and our analysis may therefore not capture possible non-linear effects. Fifth, the smaller sample size of the accelerometer-derived GWAS limits the number of available instrumental variables and may reduce statistical power. Sixth, as an inherent limitation of two-sample MR, our study cannot directly elucidate the biological mechanisms linking insomnia to osteoarthritis, however, we present a statistically robust analysis that is highly relevant to the current state of the field and may help guide future research directions; the mediation analysis identifying BMI as a partial mediator provides preliminary mechanistic insight. Future experimental and longitudinal studies are warranted to validate and further explore the causal pathways identified here. Seventh, although multiple sensitivity analyses, including MR-Egger, weighted median, and MR-PRESSO, were performed, the possibility of residual horizontal pleiotropy cannot be completely excluded.

In conclusion, our bidirectional MR analysis supports a potential causal effect of genetic predisposition to insomnia on osteoarthritis risk and provides evidence for a genetic causal effect in the reverse direction. These findings suggest a causal association between insomnia and osteoarthritis, whereas no significant associations were observed for chronotype, morningness or sleep duration after multiple-testing correction. Interventions aimed at improving sleep may therefore be relevant for OA prevention or management. Further research is needed to clarify the underlying mechanisms, including potential circadian contributions.

## Materials and methods

### Datasets and study design

For the sleep traits, we included the following UK Biobank GWAS summary statistics: (a) insomnia consisting of 453 379 individuals [38]; (b) sleep duration consisting of 446 118 individuals [39]; (c) chronotype consisting of 449 734 individuals [40]; (d) morningness consisting of 403 195 individuals [40]. Insomnia refers to having trouble falling asleep at night, or waking up in the middle of the night, as defined in the GWAS study based on UK Biobank field 1200 [38]. We utilized the GWAS summary statistic of any insomnia symptoms (‘sometimes’ and ‘usually’) versus control (‘never/rarely’) to perform the MR between insomnia and osteoarthritis-related traits. Chronotype was defined using UK Biobank field 1180 and assessed by the question ‘Do you consider yourself to be?’ with five response options: ‘Definitely a morning person,’ ‘More a morning than an evening person,’ ‘More an evening than a morning person,’ ‘Definitely an evening person,’ or ‘Do not know,’ which were coded as 2, 1, −1, −2, and 0, respectively. The response option ‘Prefer not to answer’ was treated as missing and excluded [40]. Morningness refers to the dichotomous morning person phenotype with answers of ‘Definitely a “morning” person’ and ‘More a “morning” than “evening” person’ [40]. Sleep duration refers to the continuous phenotype with an answer of the question of ‘About how many hours sleep do you get in every 24 h?’ [39]. The genetic signals for the sleep traits were validated in a subset of 92 644 participants with accelerometer data. Since all these data herein were publicly available, no ethical approval was required in this work.

For osteoarthritis, we used the summary statistics from the latest GWAS meta-analysis conducted by the Genetics of Osteoarthritis (GO) consortium, excluding UK Biobank participants to avoid sample overlap [17]. This dataset comprised of up to 1 962 069 individuals, including 489 975 osteoarthritis cases of predominantly European ancestry, across 11 osteoarthritis-related traits. As the genetic heritability estimates for sleep-related biological processes are much lower in hand osteoarthritis, spine osteoarthritis, thumb osteoarthritis and finger osteoarthritis [1], in the present study, we focused on knee osteoarthritis, hip osteoarthritis, knee and/or hip osteoarthritis, TKR, THR, total joint replacement (TJR), and osteoarthritis at any site. Moreover, to avoid inflation of type I error induced by overlapping population, a GWAS meta-analysis for these seven osteoarthritis phenotypes on individuals excluding UK biobank population were performed. A detailed overview of case numbers, total sample sizes, and SNP counts for each phenotype is provided in Table 1.

**Table 1:** Detailed information of GWAS summary statistics used in this study.

Phenotypes	Sample size (case)	SNP	Reference
Insomnia	453 379 (345022)	14 661 601	PMID: 30804566
Sleep duration	446 118	14 661 601	PMID: 30846698
Chronotype	449 734	11 977 112	PMID: 30696823
Morningness	403 195	11 977 377	PMID: 30696823
Osteoarthritis at any site	1 536 427 (404788)	24 085 836	PMID: 40205036
Hip osteoarthritis	795 156 (80233)	19 230 062	PMID: 40205036
Knee osteoarthritis	949 593 (145805)	23 655 753	PMID: 40205036
Hip/knee osteoarthritis	912 834 (172808)	23 705 742	PMID: 40205036
TJR	497 644 (48875)	11 689 107	PMID: 40205036
THR	675 390 (34674)	11 633 904	PMID: 40205036
TKR	651 543 (33536)	11 656 025	PMID: 40205036

Note: TJR, total joint replacement; THR, total hip replacement; TKR, total knee replacement; GWAS, genome-wide association study; PMID, PubMed unique identifier.

We performed bi-directional two-sample MR analyses to explore the causal effects between four sleep traits (insomnia, sleep duration, chronotype and morningness) and seven osteoarthritis-related traits (osteoarthritis at any site, hip osteoarthritis, knee osteoarthritis, hip/knee osteoarthritis, TJR, THR, TKR).

Since all these data herein were publicly available, no ethical approval was required in this work.

### Selection of instrumental variables

A genome-wide significant threshold of *P* < 5 × 10^−8^ was used to filter the significant SNPs for both sleep traits and osteoarthritis traits to be used as instrumental variables (IVs). The clump function in PLINK software [41] was used to include only variants that were independent based on linkage disequilibrium (LD) using a threshold of R^2^ < 0.001 within 10 kb base pair window using the European-based 1000 Genome Project phase 3 release reference panel [42]. To quantify the strength of instrumental variables, we calculated the F-statistic for each IV of sleep traits and osteoarthritis traits. The F-statistic was defined as *beta^2^/se^2^*, where *beta* is the effect size estimate from GWAS summary statistics, and *se* is its standard error. To avoid weak IVs effects, we removed the IVs with an F-statistic less than 10. Furthermore, if any IV was not present in the outcome trait, an LD-based proxy with R^2^ > 0.8 was included instead. To test if there is any genetic confounder and assess potential pleiotropy with genetic confounder, we evaluated associations of the instrumental SNPs with negative control outcomes including hair color and handedness.

### Estimation of causal effect and sensitivity analysis

Following the STROBE-MR guidelines [43], we performed bi-directional two-sample MR analyses between genetic predisposition to four sleep traits and seven osteoarthritis traits. Four methods were used to estimate the causal effects: random-effect IVW [44], weighted median [45], MR-Egger [46]. These different methods provide validation evidence under different conditions. IVW is the main analysis and the others are to sensitivity analyses. Moreover, we assessed heterogeneity using I^2^, a measure based on Cochran’s Q-statistics that is more interpretable and independent of the number of studies [47]. Evidence for heterogeneity, defined as I^2^ > 50, implies that some IVs may have the effect on the outcome through pathways other than the exposure, potentially violating the exclusion restriction assumption. The intercept of MR-Egger methods was used to test the horizontal pleiotropy and MR-PRESSO was used to detect potential outliers and correct the IVW estimate for these [48]. Additionally, we performed MVMR analysis [49] to estimate the direct causal effect of each sleep trait on osteoarthritis risk while simultaneously accounting for other traits (BMI and MDD) as potential confounders, thereby exploring possible mediation effects among the sleep traits. Finally, to objectively evaluate the role of sleep behaviour in osteoarthritis risk, we performed an additional MR analysis using accelerometer-derived sleep phenotypes from the UK Biobank, including diurnal inactivity, least-active 5 h (L5) timing, most-active 10 h (M10) timing, number of sleep episodes, sleep duration, sleep efficiency, and sleep midpoint, against seven osteoarthritis outcomes. Details of definition of accelerometer-derived sleep phenotypes can be found in a GWAS publication [50].

### Statistical analysis

MR analyses were performed using the R packages ‘TwoSampleMR’ [51, 52]. Forest plots and scatter plots were generated using ‘ggplot2’ R package [53]. PLINK version 1.9 was used to perform the clumping for the IVs. R package ‘LDlinkR’ [54] was used to look for proxies for missing IVs in the outcome summary statistics. To correct the P-values for multiple testing, Benjamin-Hochberg false discovery rate (FDR) was performed across all combinations of sleep traits and osteoarthritis-related traits and the significance of causal association was set to FDR < 0.05 [55]. All statistical analyses were conducted in R version 4.1.1 (https://www.R-project.org/).

## Supplementary Material

ddag043_HMG_26_00072_R1_Chen_supplementary_tables

## Data Availability

GWAS meta-analysis summary statistics are available via the GWAS Catalog (https://www.ebi.ac.uk/gwas/).
